# Peoples’ attitude toward COVID-19 vaccine, acceptance, and social trust among African and Middle East countries

**DOI:** 10.34172/hpp.2021.21

**Published:** 2021-05-19

**Authors:** Nasim Asadi Faezi, Pourya Gholizadeh, Moussa Sanogo, Amadou Oumarou, Maad Nasser Mohamed, Yacouba Cissoko, Mamadou Saliou Sow, Bakary Sayon Keita, Youssouf AG Mohamed Baye, Pasquale Pagliano, Patassi Akouda, Sid'Ahmed Soufiane, Akory Ag Iknane, Mamadou Oury Safiatou Diallo, Zakaria Gansane, Barkat Ali Khan, Şükran Köse, Hamid Allahverdipour, Khudaverdi Ganvarov, Mariam Soumaré, Mohammad Asgharzadeh, Sounkalo Dao, Hossein Samadi Kafil

**Affiliations:** ^1^Research Center for Pharmaceutical Nanotechnology, Tabriz University of Medical Sciences, Tabriz, Iran; ^2^Drug Applied Research Center, Tabriz University of Medical Sciences, Tabriz, Iran; ^3^Faculty of Pharmacy USTTB, Faculty of Medicine and Odonto Stomatology, University of Science, Technics and Technology of Bamako, Bamako, Mali; ^4^Faculte des sciences la santé de l universite Dan Dicko DanKoulodo de Maradi, Niger; ^5^Service des maladies infectieuses et tropicales de l’hôpital général peltier, Djibouti; ^6^Faculty of Medicine and Odonto Stomatology (FMOS), USTTB, University of Science, Technics and Technology of Bamako, Bamako, Mali; ^7^Service des Maladies Infectieuses, Hôpital National Donka, CHU Conakry, Centre de Recherche et de Formation en Infectiologie de Guinée (CERFIG), Guinea; ^8^Department of Medicine and Medical Specialities/Infectious Disease Unit of Fousseyni Daou Hospital, Kayes, Mali; ^9^Departement of Medicine, University of Salerno, Salerno, Italy; ^10^CHU Sylvanus Olympio, Universitie delome, Togo; ^11^Faculte de Medecine de Nouakchott, Muritanie; ^12^Institut National de Santé Publique, Bamako, Mali; ^13^Burkinabé Observatory for Healthcare Quality and Safety, Ouagadougou, Burkina Faso; ^14^Department of Pharmaceutics, Faculty of Pharmacy, Gomal University, Dera Ismail Khan, Pakistan; ^15^Department of Infectious Diseases and Clinical Microbiology, University of Health Sciences, Tepecik Training and Research Hospital, İzmir, Turkey; ^16^Research Center of Psychiatry and Behavioural Sciences and Department of Health Education and Promotion, Tabriz University of Medical Sciences, Tabriz, Iran; ^17^Department of Microbiology, Baku State University, Baku, Azerbaijan

**Keywords:** COVID-19, SARS-CoV-2, Surveys and Questionnaires, Vaccination

## Abstract

**Background:** To end the COVID-19 pandemic, a large part of the world must be immune to the virus by vaccination. Therefore, this study aimed to gauge intent to be vaccinated against COVID-19 among ordinary people and to identify attitudes towards vaccines and barriers for vaccine acceptance.

**Methods:** The study population comprises 1880 people residing in different countries that answer a prepared questionnaire. The questionnaire topics are demographics, historical issues, participants’ attitudes and beliefs regarding vaccines, concerns, and vaccine hesitancy.

**Results:** Attitudes and beliefs relating to vaccines in general, and the COVID-19 vaccine, were ascertained. Overall, 66.81% of the contributors would like to be vaccinated against COVID-19, while %33.19 did not intend to be vaccinated. Reasons for COVID-19 vaccine hesitancy included concern regarding vaccine side effects, fear of getting sick from the uptake of the vaccine, and the absence of accurate vaccine promotion news. Individuals with higher education believe that India (68.6%) produces the best vaccine (*P* <0.001), while healthcare workers think the Chinese vaccine (44.2%) is the best (*P* =0.020). Individuals with higher education have not been vaccinated, not be healthcare workers, and females were the most contributors to effective of the vaccine in reducing mortality from COVID-19 disease.

**Conclusion:** Given the degree of hesitancy against COVID-19 vaccination, a multifaceted approach to facilitate vaccine uptake that includes vaccine education, behavioral change strategies, and health promotion, is paramount.

## Introduction

COVID-19, a disease caused by severe acute respiratory syndrome coronavirus 2 (SARS-CoV-2), has spread worldwide and has been declared a pandemic with more than 130 million infected and 3 million related deaths.^1-3^ Numerous clinical trials have been conducted to investigate potential treatments for COVID-19. To bring this pandemic to an end, a large part of the world must be immune to the virus.^4^ The safest way to achieve this is to use a vaccine.^5,6^ The COVID-19 pandemic has led to tremendous advances in vaccine production at an extraordinary scale, and the LSHTM VaC (London School of Hygiene & Tropical Medicine Vaccine) tracker provides a clear testimony of this progress. COVID-19 vaccine production was much faster than other vaccines. In less than a year, several vaccines have been declared successful and approved for use in some countries.^7^ However, over the past few decades, the anti-vaccine or anti-vaccination movement has taken root in Europe and the United States.^8,9^ The anti-vaccine movement, which encourages vaccine skepticism, has emerged as a major public health problem, topping the list of global health threats.^10^ Immediately after the announcement of COVID-19 as a pandemic, countless conspiracy theories were shared on social media.^11-13^ According to the Strategic Advisory Group of experts on Immunization (SAGE), vaccine hesitancy is a term used to describe delays in accepting or refusing vaccination despite the availability of vaccination services.^14^ Factors that affect attitudes toward vaccination acceptance include satisfaction, convenience, and reliability.^14,15^ Complacency indicates a low understanding of the disease risk; hence, vaccination was considered unnecessary. Confidence refers to trust in vaccine safety, effectiveness, as well as the competence of health care systems. Convenience involves the availability, cost-effectiveness and delivery of vaccines in a comfortable condition.^15^ The complex nature of vaccine-induced motivations can be analyzed using the epidemiologic triad of environmental, causative, and host factors.^16,17^ Environmental factors include public health policies, social factors, and media messages.^18-20^ The agent factors (vaccine and disease) in addition to perceived susceptibility to disease include understanding the safety and efficacy of the vaccine.^19,21,22^ Host factors also depend on knowledge, previous experience, levels of education and income.^17,23^ Previous studies have shown that vaccine hesitancy is a common phenomenon worldwide with a variety of reasons behind refusing to be vaccinated.^24-26^ The most important reasons include perceived risks compared to benefits, some religious beliefs and lack of knowledge and awareness.^27-29^ As recent studies have shown, there is a strong association between the intention to receive coronavirus vaccines and perceived safety,^30^ a negative attitude toward vaccines and a reluctance to receive vaccines,^31^ and a link between religiosity and less willingness to receive COVID-19 vaccines.^32^ The above reasons can be applied in case of COVID-19 vaccine hesitancy. Studying the global impact of hesitancy on the COVID-19 vaccine - including the willingness to accept the COVID-19 vaccines - can be complex due to the multifaceted nature of the phenomenon.^14^ This requires cognitive, psychological, socio-demographic and cultural factors that contribute to vaccine hesitancy.^33-35^ Following an assessment of the scope and extent of the public health threat, an analysis of such factors is needed to address COVID-19 vaccine hesitancy.^36^ This can help guide intervention measures to establish and maintain a response to tackle this threat.^37^ Previous studies evaluating attitudes toward vaccines have shown regional diversity in understanding the safety and effectiveness of vaccinations.^25,38,39^ Despite many efforts to obtain successful COVID-19 vaccines, a major obstacle could be the vaccine’s hesitancy about approved and possible COVID-19 vaccinations.^40^ Therefore, the aim of this study was to gauge intent to be vaccinated against COVID-19 among ordinary people, and to identify attitudes towards vaccines and barriers for vaccine acceptance.

## Materials and Methods

### Participants and study administration

This study was a cross-sectional study conducted with institutionalized and non-institutionalized people contacted by the authors in social media. Therefore, the inclusion criteria were the people interested in answering the questionnaire and filled the form totally and correctly. In addition, we did not have a specific grouping for the participation of individuals, and various people have entered the study. The exclusion criteria were the double-filled forms. The study population comprises 1880 people residing in different countries, with different ages, who have been vaccinated or not with the COVID-19 vaccine. For preparing the questionnaire, questions were designed based on the research background, review of articles, and interviews with 20 microbiologists and infectious specialists of Tabriz University of Medical Sciences, and 25 questions were finally approved. The answer options were “Yes,” or “No,” Patients who stated “No” were prompted to indicate a reason.

The content validity ratio (CVR) was measured by asking questions of “Essential”, “Useful but not essential,” and “Not necessary” for each item of the questionnaire. The minimum value of CVR for each question was considered as 0.42 according to Lawshe’s study.^41^ The content validity index (CVI) for the whole questionnaire was calculated. The CVR of the questionnaire was adjusted at 0.79, which was accepted for questionnaire.^41^ The reliability was measured by Cronbach’s alpha, which was adjusted at 0.8.

A questionnaire was prepared by Google Doc (https://docs.google.com) in 5 different languages, including Persian, English, French, Arabic, and Istanbul. The questionnaire was distributed to different people from other countries via email or social media from February 15, 2021, through April 15, 2021. Participants finally registered their answers by clicking the submit button.

### Measures

*Survey items are included:*

Demographics of gender, age, country of residence, members of the health care system, and education were assessed.

*Historical issues:*

Participants were asked about their background disease and history of COVID-19 disease and asked about their history of vaccination against COVID-19 disease.

*Participants’ attitudes and beliefs regarding vaccines:*

Participants were asked about the impact of vaccination on recent epidemic control and reducing mortality and the country that produces the vaccine that works best.

*Concerns:*

Participants were asked about their concern for the early preparation of the vaccine in terms of safety.

*Vaccine Hesitancy:*

Participants were asked about their willingness to be vaccinated against the COVID-19 vaccine and asked their reasons for not getting the COVID-19 vaccine.

### Statistical analysis

IBM SPSS Statistics software, version 20.0 (IBM SPSS Statistics corp., Armonk, USA) and GraphPad Prism, version 8.0 (GraphPad Software, Inc., San Diego, California USA) was used for statistical analysis. Continuous covariates were summarized by mean (±SD), and categorical covariates were summarized by count (%). Categorical covariates were compared between the “Yes” and “No/Unsure” COVID-19 Vaccine groups using Chi-square or Fisher exact tests. In addition, Spearman’s rank correlation coefficient was calculated for correlation of categorical covariates. Significance was set at alpha= 0.05.

## Results

### Participant’s demographics

**Figure 1:**
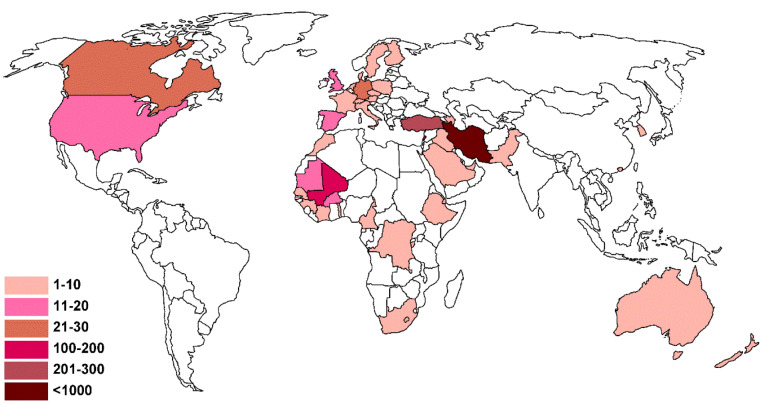
The range of participants from each country.

The total number of participants in this study was 1880 individuals who were contributed from 42 different countries. One unique individual filled all questionnaires. The range of individuals contributed from different countries was 1 to 1126, and the most frequent contributors were from Iran (1126 individuals, 59.89%), followed by Turkey (211 individuals, 11.22%), Mali (160 individuals, 8.51%), and Lebanon (117 individuals, 6.22%). The range of participants from each country is shown in the following map (Figure 1). The age range of contributors was from under 20 years old to over 60 years old, of which 54.15% were female, and 45.85% were male. The education level of contributors was from high school until Doctorate. The frequency of answers to the desired questions is shown in Table 1.

### Historical issues of participants

Overall, the contributors affected by COVID-19 disease were 21.70%, and vaccinated against COVID-19 were 6.12%. The contributors carried any background disease were 12.98%. In addition, 35.48% of the contributors work in their country’s healthcare system, which 25.33% of them have been affected by COVID-19, and 13.49% of them have been vaccinated against COVID-19 disease. Furthermore, 66.81% of the contributors would like to be immunized against COVID-19. The ages 20-40 years old were the most affected by COVID-19 (60.8%, *P* = 0.010) and the most of the individual affected was not vaccinated against COVID-19 (94.1%, *P* = 0.001). The ages 40-60 years were the most individuals who have any background diseases (49.2%, *P* < 0.001), of which 86.1% of the individuals have not been vaccinated (*P* < 0.001). Furthermore, the ages 20-60 years (86.9%, *P* < 0.001) and with higher education level (MSc. and doctorate; 63.5%, *P* = 0.016) were the most contributors that have been vaccinated, and individuals with background diseases were the lowest (29.6%, *P*< 0.001). Individuals between the ages of 20-40 years old have not been vaccinated with a higher education level.

### Participants’ attitudes and beliefsregarding vaccines

Females and individuals who have not been affected by COVID-19 were the most contributors that think vaccination can help control the recent epidemic (*P* < 0.05). Individuals with higher education believe that India (68.6%), USA (62.4%), UK (57.9%) and Europe (56.9%) produce the best vaccine (*P* < 0.001), while health-care workers think China (44.2%), India (43.8%), Russia (43.5%) and Cuba (34.8%) (*P* =0.020). Individuals with higher education have not been vaccinated, not be healthcare workers, and females were the most contributors to effective of the vaccine in reducing mortality from COVID-19 disease.

### Concerns and vaccine hesitancy of participants

**Figure 2:**
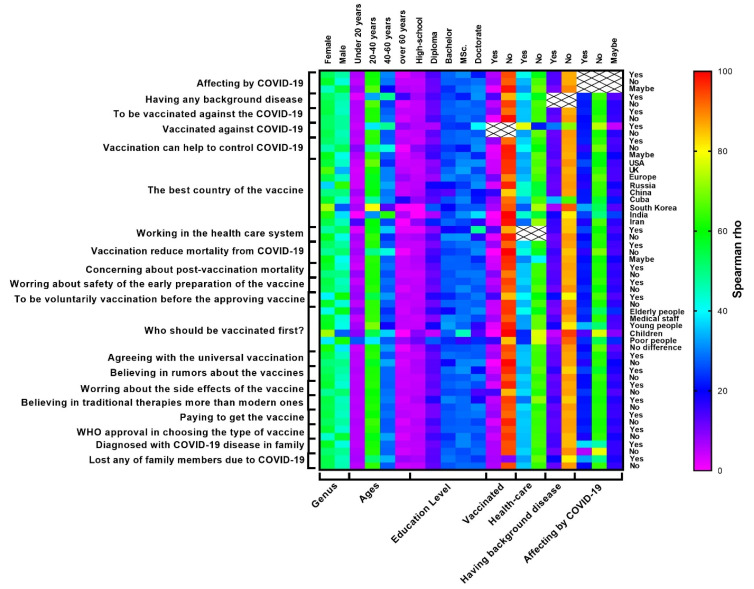
The correlation between different participant’s responses to the survey items.

Individuals with the ages 20-60 (91.3%, *P* = 0.002) have not been vaccinated (96%, *P* < 0.001), and females (56.6%, *P* = 0.007) were more concerned about the reports of post-vaccination mortality. In addition, individuals with ages of 20-60 years old (89.9%, *P* = 0.018) and who have not been vaccinated (94.8%, *P* = 0.040) were worried about the early preparation of the vaccine in terms of its safety. Most of the males (60.9%, *P* < 0.001), individuals with the ages 20-60 (86.5, *P* = 0.026), without background diseases (79.2%, *P* < 0.001) and have not been vaccinated (85.1%, *P* < 0.001) were wanted to be vaccinated voluntarily before approval of the vaccine. All the contributors were agreed that medical staff has to be vaccinated first (57.4%), followed by older people (17.6%), young people (2.4%), and children (1.2%). Individuals with high-level educations (54.55%, *P* = 0.010), who have not been affected (62%, *P* = 0.031), and have not been vaccinated (92.7%, *P* < 0.001) were agreed with the universal vaccination. Individuals with high-level educations (52.2%, *P* = 0.002), have not been affected (57.2%, *P* = 0.021), have any background disease (83.6%, *P* = 0.007), and have not been vaccinated (96.7%, *P* = 0.001) were believed rumors such as changes in the human genome in the vaccine. Females (56.5%, *P* = 0.002) and individuals that have not been vaccinated (97.3%, *P* < 0.001) were worried about the side effects of vaccines. Individuals with a high level of education (46.2%, *P* < 0.001), have not no background disease (84%, *P* = 0.033), and do not work in the healthcare system (71%, *P* = 0.001) were believed in traditional therapies more than modern ones. Individuals with the ages 20-60 years old (89.3%, *P* = 0.052), with high education levels (57.7%, *P* = 0.049), and who have not been vaccinated (91.8%, *P* < 0.001) would like to pay to get the vaccine. For all the individuals in any group properties, WHO approval was not crucial for choosing the type of vaccine except females (56.2%, *P* < 0.001). Females (57.5%, *P* = 0.008), individuals have been affected (46.5%, *P* < 0.001), have any background disease (85.4%, *P* = 0.008) has anyone in their family been diagnosed with COVID-19 disease. Individuals with the ages 40-60 years old (85.6%, *P* < 0.001), have not been affected (49.2%, *P* < 0.001) and with any background disease (80.7%, *P* = 0.002) have lost any of their family members due to COVID-19 disease. Figure 2 shows the correlation between different participant’s answers to the questions.

## Discussion

In the context of the current COVID-19 crisis, this study evaluates the intention to receive the COVID-19 vaccine in a diverse sample of individuals. Our study sample is ethnically more diverse than previous studies. Over 1880 people from 42 different countries participated in this study, which is critical to addressing ethnic differences in the prevalence, morbidity, and mortality of COVID-19. Approximately one-third (33%) of our participants were hesitant to receive the COVID-19 vaccine, and the majority (67%) intended to receive the COVID-19 vaccine. Our study was conducted from February through April 2021, when the incidences of COVID-19 and mortality rates were high in most parts of the world and at the dawn of vaccine distribution.

Various studies evaluating people’s attitudes toward the COVID-19 vaccine before vaccine distribution have reported different findings compared to our present study. However, in our study, the opinions of people from different countries were also variant. A United States poll found that only 33% of respondents were optimistic about receiving the COVID-19 vaccine. Similarly, a cross-sectional survey of 991 participants in the United States in 2020 reported that only half of the participants intended to be vaccinated against COVID-19.^42^ The results of these studies may differ from our study, as participants in previous studies are often from American countries. In addition, these studies were conducted in 2020 when the COVID-19 vaccine was hypothetical and fewer studies had been done on vaccination.

Similarly, early findings of vaccination intent may not reflect current vaccination intent. Compared to the United States, variable results have been reported in other countries. In a national survey of 3541 participants in China, 28.7% of people had a definite desire and 54.6% of a possible desire to be vaccinated.^43^ Our study shows significant degrees of vaccine safety compared to these international studies, as most people (67%) intend to receive the COVID-19 vaccine. These very different reported intentions for vaccination are probably due to differences in beliefs and social factors by the nation. For example, participants, primarily African Americans, experienced a history of medical distrust due to racial discrimination and were less likely to be vaccinated against COVID-19.

It is critical to Identify factors associated with vaccination hesitancy, enabling health professionals to develop strategic approaches to vaccine education among patients, predominantly ethnic minority patients who suffer from a disproportionate burden of COVID-19 related morbidity and mortality. Our data reported the statistical trend of financially resilient Africans who may be less likely to receive the vaccine, indicating that efforts to alleviate vaccine hesitancy should be focused on African countries.

The most common reason given by participants for avoiding COVID-19 vaccination is uncertainty and mistrust about the vaccine produced, which is dissimilar with previous vaccination studies.^44,45^ Increasing the level of transparency about vaccine safety may be an effective strategy to increase the acceptance of public vaccination against COVID-19.

The second most common reason for refusing the COVID-19 vaccine concerns the side effects of the vaccine, which can be promoted by increasing clinical studies and further studies on short-term and long-term side effects. Countries that have more confidence in the accuracy of side effects reports are more likely to get vaccinated. For instance, in a recent study, Turkish indigenous people who are more confident in their country are less reluctant to receive the vaccine. Contrary to the results of a recent study, the second reason for avoiding vaccination in other studies was the lack of recommendation from a trusted physician for vaccination.^46^ In another study, most respondents (85%) identified their physician as a reliable source of information about COVID-19 vaccination.

The third most common reason for hesitancy about vaccination is the early preparation of the COVID-19 vaccine, which is not approved in some cases. Significant researches have shown that public concerns about the safety profile of vaccines and vaccine side effects are among the essential variables influencing vaccination decisions, especially for newly developed vaccines.^47-49^ For instance, in a telephone-based interview (1155 people), approximately 13% of participants reported intentions to delay vaccination until further confirmation of side effects in others, while 17% stated that they did not intend to vaccinate.^50^ In another large study, 59% of participants intended to delay vaccination because of concerns about side effects and safety profile.

Acknowledging the reasons for avoiding vaccination provided by the laypersons, in addition to assessing health literacy and vaccination literacy of laypersons, is essential for adopting highly informative, effective vaccine campaigns and emphasizing public insurance in vaccine safety.

Our study also has its limitations. First, we used a convenience sample, so the results may not fully reflect the general attitudes of the study population. Second, the sample size and diversity of the countries participating in our study may not be underpowered. However, significant results and statistical trends show that the sample size was adequate. The strength of this study is that the timing of the survey corresponds to the peak time of the pandemic. In addition, we surveyed individuals about the intention to vaccinate when vaccines are emergently authorized and prepared to distribute to frontline healthcare workers, which is particularly relevant to the findings. Furthermore, the demographic composition of our survey team is diverse. Given the disproportionate burden of COVID-19 in minorities and underserved populations, our results may be particularly useful in informing vaccination enhancement strategies in these target communities.

## Acknowledgments

We thank all participants and people who helped us to distribute questioners and collecting data.

## Funding

This study was supported by Tabriz University of Medical Sciences with Grant number 67235.

## Competing interests

Hamid Allahverdipouris Editor-in-Chief of *Health Promotion Perspectives*. Other authors declare no competing interests.

## Ethical approval

This study was approved by the local ethic committee with registration number IR.TBZMED.REC.1399.1071. Participation in the study was voluntary, and all data were collected according to the Helsinki declaration. All information of the participants is confidential and cannot be provided for any other commercial or scientific use.

## Authors’ contributions

All authors had participation in data collection, data analysis, manuscript preparation and final proof of the manuscript. First and second authors had equal participation in this study.

**Table 1:** Patient’s characteristics and survey items

	**Frequency**	**Percent**
Gender		
Female	1018	54.15
Male	862	45.85
Age		
Under 20	105	5.59
20-40	1167	62.07
40-60	524	27.87
Over 60	84	4.47
Education level		
High school	95	5.05
Diploma	231	12.29
Bachelor	506	26.91
MSc	529	28.14
Doctorate	519	27.61
Have you ever been affected by COVID-19?		
Yes	408	21.70
No	1170	62.23
Maybe	302	16.06
Do you have any background disease?		
Yes	244	12.98
No	1636	87.02
Do you want to be vaccinated against the COVID-19 disease?		
Yes	1256	66.81
No	624	33.19
Have you been vaccinated against COVID-19 disease?		
Yes	115	6.12
No	1765	93.88
Do you think that vaccination can help to control the recent epidemic?		
Yes	1123	59.73
No	99	5.27
Maybe	658	35.00
Which country do you think produces the best vaccine?		
USA	682	36.28
UK	147	7.82
Europe	234	12.45
Russia	186	9.89
China	199	10.59
Cuba	23	1.22
South Korea	15	0.80
India	16	0.85
Iran	198	10.53
Missing	180	9.57
Do you work in the health care system of the country?		
Yes	667	35.48
No	1213	64.52
Do you think that vaccination is effective in reducing mortality from COVID-19 disease?		
Yes	1224	65.11
No	85	4.52
Maybe	571	30.37
Do reports of post-vaccination mortality cause you more concern?		
Yes	1161	61.76
No	719	38.24
Are you worried about the early preparation of the vaccine in terms of its safety?		
Yes	1135	60.37
No	745	39.63
Do you want to be vaccinated voluntarily before the vaccine is approved for mass production? (Human phase of vaccine production)		
Yes	289	15.37
No	1591	84.63
In your opinion, who should be vaccinated first?		
Elderly people	316	16.81
Medical staff	1030	54.79
Young people	43	2.29
Children	22	1.17
Poor people	26	1.38
No difference	357	18.99
Missing	86	4.57
Do you agree with the universal vaccination against COVID-19 disease?		
Yes	1549	82.39
No	331	17.61
Do you believe in rumors such as changes in the human genome by vaccines?		
Yes	519	27.61
No	1361	72.39
Are you worried about the side effects of the Corona vaccine?		
Yes	1335	71.01
No	545	28.99
Do you believe in traditional therapies more than modern ones?		
Yes	431	22.93
No	1449	77.07
Would you like to do this if you had to pay to get the vaccine?		
Yes	1146	60.96
No	734	39.04
Is WHO approval essential for you in choosing the type of vaccine?		
Yes	1572	83.62
No	308	16.38
Has anyone in your family been diagnosed with COVID-19 disease?		
Yes	929	49.41
No	936	49.79
Missing	15	0.80
Have you lost any of your family members due to COVID-19 disease?		
Yes	244	12.98
No	1636	87.02
